# Evaluation of 3-l- and 3-d-[^18^F]Fluorophenylalanines as PET Tracers for Tumor Imaging

**DOI:** 10.3390/cancers13236030

**Published:** 2021-11-30

**Authors:** Felicia Krämer, Benedikt Gröner, Chris Hoffmann, Austin Craig, Melanie Brugger, Alexander Drzezga, Marco Timmer, Felix Neumaier, Boris D. Zlatopolskiy, Heike Endepols, Bernd Neumaier

**Affiliations:** 1 Institute of Radiochemistry and Experimental Molecular Imaging, Faculty of Medicine and University Hospital Cologne, University of Cologne, 50937 Cologne, Germany; maximiliane-felicia.kraemer@uk-koeln.de (F.K.); b.groener@fz-juelich.de (B.G.); ch.hoffmann@fz-juelich.de (C.H.); a.craig@hzdr.de (A.C.); felix.neumaier@uk-koeln.de (F.N.); boris.zlatopolskiy@uk-koeln.de (B.D.Z.); heike.endepols@uk-koeln.de (H.E.); 2 Nuclear Chemistry (INM-5), Institute of Neuroscience and Medicine, Forschungszentrum Jülich GmbH, Wilhelm-Johnen-Straße, 52428 Jülich, Germany; m.brugger@fz-juelich.de; 3 Department of Nuclear Medicine, Faculty of Medicine and University Hospital Cologne, University of Cologne, 50937 Cologne, Germany; alexander.drzezga@uk-koeln.de; 4 German Center for Neurodegenerative Diseases (DZNE), 53127 Bonn-Cologne, Germany; 5 Molecular Organization of the Brain (INM-2), Institute of Neuroscience and Medicine, Forschungszentrum Jülich GmbH, Wilhelm-Johnen-Straße, 52428 Jülich, Germany; 6 Center for Neurosurgery, Faculty of Medicine and University Hospital Cologne, University of Cologne, 50937 Cologne, Germany; marco.timmer@uk-koeln.de; 7 Max Planck Institute for Metabolism Research, 50931 Cologne, Germany

**Keywords:** PET, nuclear medicine, radiotracer, orthotopic xenograft model, [^18^F]FPhe, glioblastoma

## Abstract

**Simple Summary:**

The early detection and treatment of malignant brain tumors can significantly improve the survival time and life quality of affected patients. Whereas positron emission tomography (PET) with *O*-(2-[^18^F]fluoroethyl)tyrosine ([^18^F]FET) offers improved diagnostic accuracy compared to other imaging methods, there is still a need for PET tracers with better tumor-specificity. A higher protein incorporation rate, as well as a higher affinity for the amino acid transporter LAT1, could provide probes with superior image quality compared to [^18^F]FET. The aim of the present study was a preclinical evaluation of the two enantiomeric phenylalanine (Phe) analogues, 3-l- and 3-d-[^18^F]fluorophenylalanine ([^18^F]FPhes), as possible alternatives to [^18^F]FET. Based on promising in vitro evaluation results, the radiolabeled amino acids were studied in vivo in two subcutaneous and one orthotopic rodent tumor xenograft models using µPET. The results show that 3-l- and 3-d-[^18^F]FPhe enable high-quality visualization of tumors with certain advantages over [^18^F]FET, making them promising candidates for further preclinical and clinical evaluations.

**Abstract:**

Purpose: The preclinical evaluation of 3-l- and 3-d-[^18^F]FPhe in comparison to [^18^F]FET, an established tracer for tumor imaging. Methods: In vitro studies were conducted with MCF-7, PC-3, and U87 MG human tumor cell lines. In vivo µPET studies were conducted in healthy rats with/without the inhibition of peripheral aromatic l-amino acid decarboxylase by benserazide pretreatment (*n* = 3 each), in mice bearing subcutaneous MCF-7 or PC-3 tumor xenografts (*n* = 10), and in rats bearing orthotopic U87 MG tumor xenografts (*n* = 14). Tracer accumulation was quantified by SUV_max_, SUV_mean_ and tumor-to-brain ratios (TBrR). Results: The uptake of 3-l-[^18^F]FPhe in MCF-7 and PC-3 cells was significantly higher relative to [^18^F]FET. The uptake of all three tracers was significantly reduced by the suppression of amino acid transport systems L or ASC. 3-l-[^18^F]FPhe but not 3-d-[^18^F]FPhe exhibited protein incorporation. In benserazide-treated healthy rats, brain uptake after 42–120 min was significantly higher for 3-d-[^18^F]FPhe vs. 3-l-[^18^F]FPhe. [^18^F]FET showed significantly higher uptake into subcutaneous MCF-7 tumors (52–60 min p.i.), while early uptake into orthotopic U87 MG tumors was significantly higher for 3-l-[^18^F]FPhe (SUV_max_: 3-l-[^18^F]FPhe, 107.6 ± 11.3; 3-d-[^18^F]FPhe, 86.0 ± 4.3; [^18^F]FET, 90.2 ± 7.7). Increased tumoral expression of LAT1 and ASCT2 was confirmed immunohistologically. Conclusion: Both novel tracers enable accurate tumor delineation with an imaging quality comparable to [^18^F]FET.

## 1. Introduction

Gliomas are the second most common primary brain tumors in adults after meningiomas [1]. Despite intense research efforts and the development of new treatment approaches, many of these tumors are still associated with a poor prognosis and limited survival time. For example, roughly half of all glioma patients are diagnosed with glioblastoma multiforme, a particularly malignant form with median survival times of less than 1 year [2]. Therefore, there is an urgent need for techniques that facilitate early diagnosis, accurate tumor delineation, and detection of the most malignant regions for, e.g., surgery and therapy planning [2]. Whereas magnetic resonance imaging (MRI) can be used for tumor detection, the exact circumscription of brain tumors, such as gliomas with MRI, is complicated by their diffusely infiltrative character [3,4,5,6]. Positron emission tomography (PET) with radiolabeled amino acids is a promising alternative for the imaging of such tumors. This method is based on the higher protein synthesis rate and/or expression of certain amino acid transporters in most cancer cells [6,7]. In particular, the vast majority of neoplastic tissues, such as gliomas, breast, and prostate cancers, prominently overexpress the system L amino acid transporter LAT1 [3,8,9,10] and other amino acid transporters, such as ASCT2 [11,12], which have been identified as “cancer-promoting targets” and part of the “tumor metabolome” [11,13]. This is exploited for PET-based imaging with *O*-(2-[^18^F]fluoroethyl)tyrosine ([^18^F]FET), which acts as a substrate of these transporters and accumulates preferentially in neoplastic tissues [3]. Due to the low uptake of amino acids in normal brain parenchyma, tracers such as [^18^F]FET are especially well suited for tumor imaging in the brain, where the diagnostic performance of other probes such as [^18^F]fluorodeoxyglucose ([^18^F]FDG) is low. More specifically, [^18^F]FET-PET imaging facilitates the delineation of brain tumors from surrounding healthy tissue, and from unspecific alterations such as neuroinflammation, edema, or necrosis [3]. In addition, [^18^F]FET-PET can be used to distinguish therapy-associated pseudo-progression from real tumor progression during therapy monitoring [14,15]. 

[^18^F]FET exhibits no protein incorporation, but is transiently trapped within cells [16]. This may be related to asymmetries in the intra- and extracellular substrate recognition by LAT1 [17]. Since protein biosynthesis is also upregulated in many tumors, a higher protein incorporation rate could facilitate the sustained trapping of PET tracers in tumor tissues, thereby increasing the tumor-to-background ratio (TBR) and improving the image quality. The naturally occurring amino acid l-phenylalanine (l-Phe) is an essential proteinogenic amino acid [18]. Therefore, its radiofluorination in the 3-position of the aromatic ring could provide a probe that combines high LAT1 affinity with high protein incorporation rates and might thus serve as a marker for increased protein biosynthesis [19]. In contrast, its enantiomer d-phenylalanine (d-Phe) should not be incorporated into proteins. Accordingly, its accumulation in healthy tissues should be reduced when compared to l-Phe, leading to a higher TBR. Since LAT1 shows no stereospecificity and is overexpressed in many tumor cells as mentioned above, radiofluorinated d-Phe analogs should still enable delineation of tumors with enhanced TBR while minimizing radiation exposure of healthy tissues [20]. Thus, the aim of the present study was to evaluate the potential of 3-l- and 3-d-[^18^F]fluorophenylalanine (3-l- and 3-d-[^18^F]FPhe) for tumor imaging. To this end, both compounds were prepared by alcohol-enhanced, copper-mediated radiofluorination [21] and compared with [^18^F]FET in in vitro cell uptake experiments, in vivo experiments in healthy rats, and subcutaneous and orthotopic xenograft tumor models using µPET. Since decarboxylation outside of the brain often reduces the brain delivery of radiolabeled amino acids, pretreatment with the aromatic l-amino acid decarboxylase (AADC) inhibitor benserazide was used to elucidate the impact of peripheral decarboxylation on brain uptake of the radiolabeled amino acids. 

## 2. Materials and Methods

### 2.1. Preparation of 3-l- and 3-d-[^18^F]FPhes

**Figure 1:**
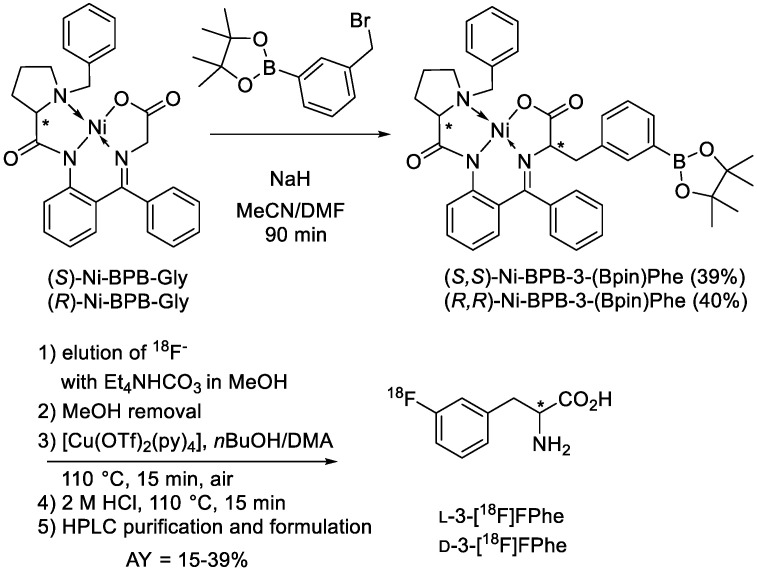
Preparation of 3-l- and 3-d-[^18^F]FPhes. AY—activity yield.

The radiolabeling precursors, (*S*,*S*)- and (*R*,*R*)-Ni-BPB-(3-Bpin)Phes [22], were prepared in 39% and 40% yield, respectively, by alkylation of the corresponding (*S*)- or (*R*)-Ni-BPB-Gly-complexes [23] with 3-(bromomethyl)phenylboronic acid pinacol ester (Figure 1).

3-l- and 3-d-[^18^F]FPhe were produced using alcohol-enhanced Cu-mediated ^18^F-fluorodeboronation [21,24] as described previously [22]. The provided activity yields [25] refer to non-decay corrected yields of the tracers formulated as ready-to-use solutions.

[^18^F]Fluoride (0.5–10 GBq) was loaded (from the male side) onto a QMA carbonate light plus cartridge (130 mg, Waters GmbH, Germany; preconditioned with 2 mL H_2_O). The cartridge was washed with MeOH (1 mL), dried with air (10 mL), and eluted (from the female side) with a solution of Et_4_NHCO_3_ (1 mg, 5.23 µmol) in MeOH (0.5 mL). If the QMA cartridge was loaded, flushed, and eluted from the female side only, a significant amount of [^18^F]fluoride sometimes remained on the resin (probably because QMA-light cartridges have a single frit on the male side but four frits on the female side). MeOH was removed within 3 min at 80 °C under reduced pressure (300–400 mbar) in a stream of argon. A solution of the respective precursor, (*S*,*S*)- or (*R*,*R*)-Ni-BPB-(3-Bpin)Phe, (7.1 mg, 10 µmol) and [Cu(OTf)_2_(py)_4_] (13.6 mg, 20 µmol) in *n*BuOH/DMA 1:2 (0.75 mL), was added, and the reaction mixture was stirred under air at 110 °C for 15 min. The mixture was concentrated at the same temperature under reduced pressure (300–400 mbar) and in a stream of argon for 5 min. Then, the residue was taken up in 2 M HCl (0.5 mL) and stirred at 110 °C for 15 min. The reaction mixture was cooled at ambient temperature for 1–2 min to about 40–60 °C, diluted with H_2_O (1 mL) and the radiolabeled product was isolated by preparative HPLC. The product fraction was concentrated under reduced pressure in a stream of argon, and the residue was taken up in saline to afford a ready-to-use solution of the corresponding tracer. The synthesis time, including purification and formulation, amounted to 90 min. Molar activities of the radiolabeled amino acids were determined from the corresponding calibration curve using HPLC (detection at 210 nm).

Analytical and preparative HPLC was performed on a Dionex Ultimate 3000 HPLC system and a DAD UV detector coupled in series with a Berthold NaI detector. The identity of ^18^F-labeled 3-FPhes was confirmed by co-injection of an authentic sample of 3-fluorophenylalanine. The UV and radioactivity detectors were connected in series, giving a time delay of 0.2–0.9 min between the corresponding responses depending on the flow rate.

Analytical HPLC. Column: Synergi Hydro-RP, 4 µm, 80 Å, 250 × 4.6 mm equipped with the appropriate SecurityGuard™ cartridge (2 × 3 mm) (Phenomenex, Aschaffenburg, Germany); eluent: 20% MeCN (0.1% TFA); flow rate: 1 mL/min. *R*_t_(3-[^18^F]FPhe): 9.4 min.

Preparative HPLC. Column: Synergi Hydro-RP, 4 μm, 80 Å, 250 × 10 mm equipped with the appropriate SecurityGuard™ cartridge (2 × 3 mm) (Phenomenex, Aschaffenburg, Germany); eluent: 10% EtOH (0.1% TFA); flow rate: 4.7 mL/min. Product fraction: 8–9 min.

### 2.2. Cell Cultures

MCF-7 breast tumor cells (DSMZ: ACC 115), PC-3 prostate tumor cells (DSMZ: ACC 465) and U87 MG glioblastoma cells (ATCC: HTB-14) were used. MCF-7 cells were cultured in minimum essential medium GlutaMAX (MEM, Gibco 41090028, Fisher Scientific GmbH, Schwerte, Germany) supplemented with 10% fetal bovine serum (FBS, Sigma-Aldrich F2442, Merck KGaA, Darmstadt, Germany), 1% penicillin/streptomycin (Gibco 115140122), 1% non-essential amino acids (NEAA, Gibco 11140050), 1% human recombinant insulin (Sigma Aldrich 91077C), and 1% sodium pyruvate (ThermoFisher 11360070, Fisher Scientific GmbH, Schwerte, Germany). PC-3 cells were cultured in F-12 K nutrient mix medium supplemented with 10% FBS and 1% penicillin/streptomycin. U-87 MG cells were cultured in MEM supplemented with 10% FBS, 1% penicillin/streptomycin, and 1% NEAA. All cell lines were cultured in cell-culture dishes (ThermoFisher 150350, Φ 100 mm) containing the culture medium (9 mL) in a humidified atmosphere of 5% CO_2_/95% air at 37 °C for 4–5 days until they reached 80–90% confluency. Cells were seeded into 12-well plates (2 × 10^5^ cells/well containing 1 mL medium) 48 h before the beginning of the cellular experiments.

### 2.3. Cellular Uptake Experiments

Two hours prior to the start of experiments, the culture medium was carefully aspirated, the cells were washed with phosphate-buffered saline (PBS, 1 mL, Gibco 10010023), and a dye exclusion test with trypan blue (Sigma Aldrich T 8154) was performed to determine cell viability and the exact cell count. The viability was always >95%. The tracer solution was prepared with serum- and amino acid-free Earle’s balanced salt solution (EBSS) at a concentration of 150 kBq/mL. PBS was removed from the wells and the tracer solution was added (1 mL/well). The cells were then incubated at 37 °C for a given time. Thereafter, the cells were washed twice with ice-cold PBS (1 mL), trypsinized and harvested. The accumulated radioactivity was measured in an automatic gamma counter (Hidex AMG version 1.4.4, Turku, Finland). Each experiment was conducted at least in triplicate. The reference tracer for all cellular uptake experiments was [^18^F]FET.

### 2.4. Cellular Inhibition Experiments 

For the inhibition experiments, U87 MG cells were used and cultured as described above. The following inhibitors, obtained from Sigma Aldrich, were used: 2-(methylamino)-2-methylpropionic acid (MeAIB) for system A, l-serine for system ASC and 2-aminobicyclo[2,2,1]heptane-2-carboxylic acid (BCH) for system L. The inhibitors were diluted with EBSS to give the desired final concentrations (150 nM, 15 nM, 1.5 nM) and added together with the tracer. The further procedures were as described above.

### 2.5. Protein Incorporation of 3-l-[^18^F]FPhe and 3-d-[^18^F]FPhe in U87 MG Tumor Cells

Cell cultivation, the determination of cell viability, and cell counts were performed as described above. The experimental medium was replaced by 1 mL of the tracer solution (150 kBq/mL), and the cells were incubated for 60 min at 37 °C. After removal of the tracer solution, the cells were washed twice with 1 mL ice-cold PBS and resuspended in 1 mL PBS. The cells were scraped off, and the cell suspension was transferred to a reaction vial (Eppendorf AG, Hamburg, Germany) and centrifuged at 2500× *g* for 5 min at ambient temperature. The supernatant was discarded, and the cell pellet was resuspended with 1 mL lysis buffer (5 mM Tris·HCl pH 7.4 + 2 mM EDTA in H_2_O). The cells were homogenized with a disperser (Ultra-Turrax^®^, Proxxon, Wecker, Luxembourg) at the highest level for 1 min at 4 °C, and the resulting homogenate was centrifuged again (30 min at 18,400× *g* and 4 °C). The supernatant was loaded onto a PD 10 cartridge (VWR International GmbH, Darmstadt, Germany) conditioned with 25 mL EBSS. The cartridge was washed with 25 mL EBSS. Consecutive fractions (1 mL each) were collected, with high molecular weight components being eluted first and measured by a gamma counter.

### 2.6. Experimental Animals

All experiments were conducted in accordance with the EU directive 2010/63/EU for animal experiments and the German Animal Welfare Act (TierSchG, 2006) and were approved by the regional authorities (LANUV, NRW; 84-02.04.2017.A288). For biodistribution studies, 6 healthy female and 2 healthy male Long Evans rats (207–734 g body weight) were used (Janvier Labs, Le-Genest-Saint-Isle, France). In total, 14 immunodeficient male Rowett Nude Rats (Crl:NIH-Foxn1rnu, Charles River; 241–506 g body weight) and 10 immunodeficient male SCID mice (C.B-Igh-1b/IcrTac-Prkdcscid, Janvier Labs, Le-Genest-Saint-Isle, France; 21–28 g body weight) were used for the tumor xenograft models.

All animals were housed in groups of up to 5 animals in individually ventilated cages (NexGen Ecoflo, cages Rat1800 or Mouse500; Allentown Inc., Allentown, NJ, USA) under controlled ambient conditions (22 ± 1 °C and 55 ± 5% relative humidity) and on a 12 h light/dark schedule (lights on from 9:00 p.m. to 9:00 a.m.). Food and water for all animals were available ad libitum. The health status of the animals was monitored daily and showed no changes throughout the experiments.

### 2.7. Biodistribution Experiments in Healthy Rats by PET

One-half of the healthy rats were injected with benserazide (15 mg/kg bodyweight i.p.) 1 h prior to tracer injection and allowed to move again freely in their cages while the other animals were anesthetized just before tracer injection. After anesthesia induction with 5% isoflurane in O_2_/air (3:7), the isoflurane concentration was reduced (to 1.5–2.5%), and the rats were placed in an animal holder (Minerve, Esternay, France) and fixed with a tooth bar in a respiratory mask. The PET scans started upon injection of the tracer into the tail vein (60.7–72.2 MBq in 0.5 mL i.v.). During the measurements, respiratory rate was monitored and maintained at around 40–60 breaths per minute by adjusting the isoflurane concentration. Core body temperature was maintained at about 37 °C by warm airflow through the animal bed. Dynamic PET scans in list mode were performed with a Focus 220 micro-PET scanner (CTI-Siemens, Knoxville, TN, USA) with a resolution at center of field of view of 1.4 mm. Data acquisition started with tracer injection and was continued for 120 min followed by a 10 min transmission scan using a ^57^Co point source. Following Fourier rebinning, data were reconstructed using an iterative OSEM3D/MAP procedure [26], including attenuation and decay correction in two different ways: (1) 28 frames (2 × 1 min, 2 × 2 min, 6 × 4 min, 18 × 5 min) for the compilation of regional time activity curves (TACs), and (2) 4 frames (4 × 30 min) for visual display. The resulting voxel sizes were always 0.38 mm × 0.38 mm × 0.79 mm. Data analysis was performed using the software VINCI [27]. Summed images were manually co-registered with a structural MR image template and Gauss filtered (1 mm FWHM), and then SUV_bw (=bodyweight)_ was determined by dividing each image by the injected dose and multiplying the result by body weight times 100. To obtain TACs, an elliptical volume of interest (VOI) was placed over the whole brain and another one over the lambdoidal crest to assess defluorination. Mean SUVs were extracted from each of the 28 frames and plotted over time.

### 2.8. Subcutaneous Tumor Models

For subcutaneous tumor xenografts, a suspension of MCF-7 (1 × 10^7^ in 75 µL matrigel; *n* = 5) or PC-3 tumor cells (1 × 10^7^ in 75 µL matrigel; *n* = 5) was injected subcutaneously in the scruff of the neck near the right shoulder of the mice. After 2 weeks the mice developed palpable tumors of about 0.5–1 cm^3^. PET scans were performed as described above with a scan time of 60 min after tracer injection (6.1–10.7 MBq in 125 μL i.v.). All PET images were normalized to the injected dose per kg body weight. Summed PET images over 0–60 min post injection were used for determining mean and maximum SUV_bw_ of the tumor, muscle (thigh), kidneys, liver, intestine, and bone. The resulting voxel sizes were 0.47 mm × 0.47 mm × 0.80 mm. The tumor volume was determined using the well-defined tumor VOI. Multi-frame files (2 × 1 min, 2 × 2 min, 6 × 4 min, 6 × 5 min) were used to generate the TACs by placing VOIs on the tumor, liver, and intestine. After completion of the measurements, the tumor-bearing mice were sacrificed by cervical dislocation under isoflurane anesthesia and the tumors were removed for immunohistochemical staining.

### 2.9. Orthotopic Tumor Model

For the orthotopic model, U87 MG glioma cells (10^5^ cells in 1 µL) were implanted stereotactically (Classic Lab Standard^TM^ Stereotaxic Instrument, Stoelting, Dublin, Ireland) under inhalation anesthesia (initial dosage of 5% isoflurane in O_2_/air (3:7), maintenance 3–4% isoflurane) into the brain of male Rowett Nude rats. The stereotactic coordinates were 0.5 mm anterior, 2.5 mm lateral, and 4.5 mm ventral from Bregma. MRI scans were performed 1, 2, and 3 weeks after tumor cell implantation to determine the size of the intracranial tumor. The measurements were performed under general anesthesia (initial dosage of 5% isoflurane in O_2_/air (3:7), maintenance 2–2.5% isoflurane) in an MRI scanner (3T Achieva^®^, Philips Healthcare, Best, The Netherlands) in combination with an 8 Channel Volumetric Rat Array (Rapid Biomedical GmbH, Rimpar, Germany). Three-dimensional T2-weighted MR images were acquired using a turbo-spin echo sequence with repetition time = 14 s, echo time = 30 ms, field of view = 60 × 60 × 60 mm^3^, and voxel size = 0.5 × 0.5 × 0.5 mm^3^.

PET scans and image reconstruction were performed as described above. The PET images were manually co-registered with the corresponding MR image and Gauss filtered (1 mm FWHM), and then the SUV_bw_ values were calculated. The SUV_max_ and SUV_mean_ values of the tumor, unaffected brain tissue, and lambdoidal crest were also calculated. Tumor volumes and TACs were generated as described above. After the experiments, the anesthetized rats were sacrificed by perfusion fixation and the brains were removed for immunohistochemical analysis.

### 2.10. Histological Staining

Cryosections of the glioma-containing brain regions were stained with Hematoxyclin and Eosin (HE). Amino acid transporters were visualized with anti-LAT1/SLC7A5-antibodies (LS-B15706, LSBio, Seattle, WA, USA) and anti-ASCT2/SLC1A5-antibodies (ABIN630342, antibodies-online, Aachen, Germany), respectively. After incubation with 4% paraformaldehyde (PFA) for 1 h, the sections were washed with PBS (Fisher BioReagents^®^, Fisher Scientific GmbH, Schwerte, Germany) before antigen retrieval was performed in autoclave sterilized 0.01 M sodium citrate buffer (pH 6.0), heated up to 97 °C for 10–15 min. Afterwards, the sections were washed again with PBS and then incubated with blocking medium (10% goat serum solution with 0.3% Triton, Vector Laboratories, CA, USA) for 1 h at room temperature before incubation with one of the described antibodies (dilution factor 1:500 for the anti-ASCT2 antibody and 1:200 for the anti-LAT1 antibody) at 4 °C overnight. On the following day, the slides were sequentially incubated with goat anti-rabbit secondary antibody (Vectastain Elite ABC kit, Vector Laboratories, CA, USA), Avidin-Biotin complex (Vectastain Elite ABC kit: Avidin and biotinylated peroxidase, Vector Laboratories, CA, USA) and finally diaminobenzidine solution (1 tablet 3,3′-diaminobenzidine + 30 µL 3% H_2_O_2_ were dissolved by vortexing in 5 mL distilled water; Sigma-Aldrich/Merck KgaA, Darmstadt, Germany). In between, the slides were washed with PBS solution and ethanol. The evaluation of the histological images was carried out using the Image J software (https://imagej.nih.gov/ij/; last accessed on 10 November 2020) at a defined threshold value of 50% for binary transformation.

### 2.11. Statistical Analysis

All statistical analyses were performed using GraphPad Prism 8.0 (GraphPad Software, San Diego, CA, USA) for Windows 7. For the cellular uptake experiments, a 2-way analysis of variance (ANOVA) was performed with the factors “cell line” and “radioligand”, “cell line” and “time-point,” as well as “radioligand” and “time-point”. For the biodistribution studies in healthy rats, 2-way ANOVAs with the factors “organ” (brain, bone) and “benserazide pre-treatment” (with and without) were performed for every time-point and radioligand. In addition, a 3-way ANOVA with the factors “organ”, “radioligand” and “benserazide-treatment” was performed for the time frame 30–60 min p.i. For the PET experiments with tumor xenograft bearing mice and rats, 3 separate 2-way mixed design ANOVA tests (for each tumor, liver, and intestine, respectively, tumor, brain, and bone) were used with the factors “radioligand” and “time-point”. One-way ANOVAs were used for the tumor-to-muscle ratio respective to tumor-to-brain ratio. All ANOVA tests were followed by Sidak’s, Tukey’s, or Dunnett’s multiple comparison procedures. Statistical significance was defined as a *p* value of less than 0.05.

## 3. Results

### 3.1. Tracer Synthesis

As described previously, 3-l- and 3-d-[^18^F]FPhes were prepared from the corresponding diastereomerically and enantiomerically pure Ni-BPB-Phe precursors (BPB: (*S*)-2-[*N*-(*N′*-benzylprolyl)amino]benzophenone) bearing a pinacol boronate moiety [22] in 15–39% activity yields (n > 10) within 90 min and with molar activities of 180–250 GBq/µmol using alcohol-enhanced Cu-mediated ^18^F-fluorodeboronation (Figure 1) [21].

### 3.2. In Vitro Cellular Uptake Studies

#### 3.2.1. Protein Incorporation Studies

**Figure 2:**
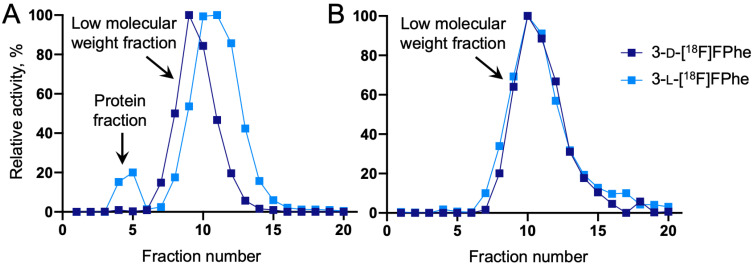
Elution profiles of 3-l-[^18^F]FPhe and 3-d-[^18^F]FPhe after (**A**) incubation with U87 MG cells for 60 min and subsequent cell lysis or (**B**) control experiments with tracer solution only (no incubation). Note that in contrast to 3-l-[^18^F]FPhe, 3-d-[^18^F]FPhe was not incorporated into proteins.

The degree of protein incorporation was examined in U87 MG cells incubated for 60 min with a solution containing either 3-l-[^18^F]FPhe or 3-d-[^18^F]FPhe. Thereafter, cells were lysed, and the soluble fractions were separated using PD10 columns. Proteins containing radiolabeled 3-l-[^18^F]FPhe were found in fractions 4 and 5, whereas free 3-l-[^18^F]FPhe and its metabolites were eluted in the late fractions 7–16 (Figure 2). The activity of the protein fraction relative to the low molecular weight fraction was approximately 20%. For 3-d-[^18^F]FPhe, no radioactivity was present in the early fractions, indicating that it was not incorporated into proteins.

#### 3.2.2. Absolute and Relative Cellular Uptake

**Figure 3:**
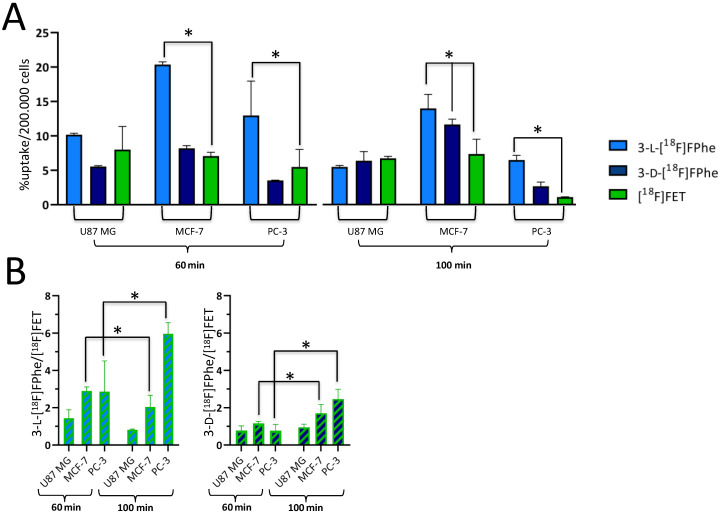
(**A**) Absolute uptake of 3-l-[^18^F]FPhe compared to 3-d-[^18^F]FPhe and [^18^F]FET in different tumor cell lines after incubation for 60 and 100 min. * indicates significant differences in cellular uptake compared to [^18^F]FET. (**B**) Relative uptake of 3-l-[^18^F]FPhe (3-l-[^18^F]FPhe/[^18^F]FET ratio) and 3-d-[^18^F]FPhe (3-d-[^18^F]FPhe/[^18^F]FET ratio). * indicates significant differences between timepoints. F(2,159) = 137.0, *p* < 0.0001 for factor “cell line”, Sidak’s post-hoc *p* < 0.05 and F(2,195) = 44.29, *p* < 0.0001, Sidak’s post hoc <0.05.

The results of the cellular uptake experiments performed with 3-l-[^18^F]FPhe, 3-d-[^18^F]FPhe and [^18^F]FET in MCF-7 (human breast adenocarcinoma), PC-3 (human prostate adenocarcinoma), and U87 MG (human glioblastoma) tumor cells are summarized in Figure 3. Specific cellular uptake in MCF-7 and PC-3 cells differed significantly between the three radiotracers (60 min: F(5,64) = 41.55, *p* < 0.0001 for factor “tracer”; 100 min: F(5,54) = 32.91, *p* < 0.0001). Post-hoc analysis revealed a significantly higher cellular uptake of 3-l-[^18^F]FPhe when compared to [^18^F]FET after 60 and 100 min of incubation in the experiments with MCF-7 (60 min: post hoc *p* < 0.0001; 100 min: post hoc *p* < 0.0001) and PC-3 (60 min: post hoc *p* < 0.0001; 100 min: post hoc *p* < 0.0001) cells (Figure 3A). Interestingly, the level of tracer uptake in U87 MG cells after 100 min of incubation was either similar to (for 3-d-[^18^F]FPhe and [^18^F]FET) or even lower than (for 3-l-[^18^F]FPhe) the level of tracer uptake measured in the same cells after 60 min of incubation. In addition, there was a significant difference in the uptake of both enantiomers after 60 and 100 min if expressed relative to the [^18^F]FET uptake (Figure 3B). In particular, the 3-l-[^18^F]FPhe / [^18^F]FET ratio after 100 min of incubation was significantly lower in MCF-7 cells (post hoc *p* = 0.0002) and significantly higher in PC-3 cells (post hoc *p* < 0.0001). The 3-d-[^18^F]FPhe / [^18^F]FET ratio after 100 min of incubation was significantly higher in both the MCF-7 (post hoc *p* < 0.0001) and PC-3 (post hoc *p* < 0.0001) cells. Thus, based on in vitro evaluation, the uptake profiles of the two enantiomeric [^18^F]FPhes in the studied tumor cell lines were superior to that of [^18^F]FET.

#### 3.2.3. Tracer Uptake Kinetics

**Figure 4:**
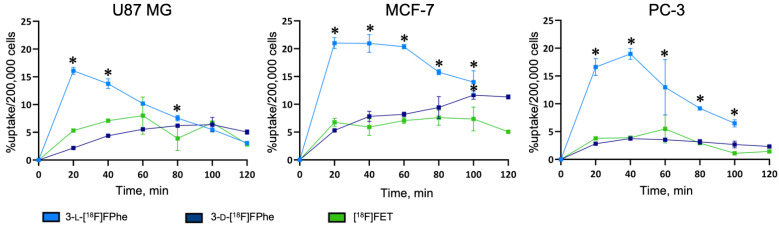
Kinetics of tracer uptake in different tumor cell lines determined at 37 °C. * indicates significant differences in cellular uptake compared to [^18^F]FET.

We next performed a more detailed analysis of tracer uptake kinetics in the different tumor cell lines. As displayed in Figure 4, 3-l-[^18^F]FPhe showed a rapid uptake in all tested cell lines and, by far, the highest overall uptake examined over the whole time course.

In contrast, 3-d-[^18^F]FPhe and [^18^F]FET showed a lower uptake that was similar for both tracers and rapidly reached a plateau. After 100 min, the differences between the various tracers were insignificant for all tested cell lines.

#### 3.2.4. Competitive Inhibition Studies

**Figure 5:**
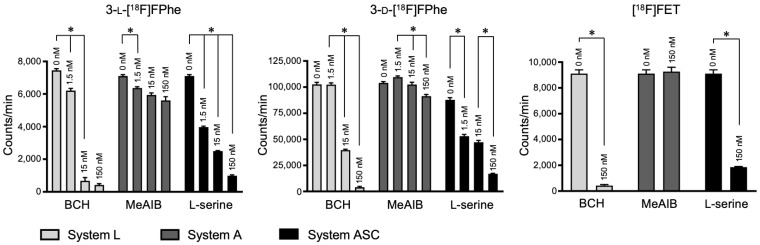
Uptake of 3-[^18^F]FPhes and [^18^F]FET in U87 MG cells in the presence of increasing concentrations of different specific amino acid transporter inhibitors (0, 1.5, 15 and 150 nM). Inhibitors: BCH for system L, B^0^, and B^0,+^, MeAIB for system A and l-serine for system ASC (uptake expressed as counts per minute, mean ± SD, *n* = 3). * indicates significant differences between experiments with different inhibitor concentrations.

To identify the transport systems responsible for cellular accumulation of the tracers, the uptake in U87 MG cells was measured in the absence and presence of different specific amino acid transporter inhibitors. An effective inhibition of tracer uptake was observed if the most important amino acid transport systems L or ASC were blocked with 2-aminobicyclo[2,2,1]heptane-2-carboxylic acid (BCH) or l-serine, respectively (Figure 5). Especially at higher concentrations of these inhibitors, the uptake of all three tracers was significantly reduced, suggesting that they were mainly transported into the cells via the L and ASC transport system. The fact that both enantiomers showed a similar response to the competitive inhibition of system L confirms the non-stereospecific transport via system L transporters. A much lower inhibitory effect on uptake was observed for 3-l- and 3-d-[^18^F]FPhe if system A was blocked with MeAIB. [^18^F]FET uptake was not affected by inhibition of system A. The high uptake of 3-[^18^F]FPhes in the tumor cell lines prompted us to investigate the tracers in animal models.

### 3.3. In Vivo Studies

#### 3.3.1. Brain Distribution and Skull Bone Uptake in Healthy Rats

**Figure 6:**
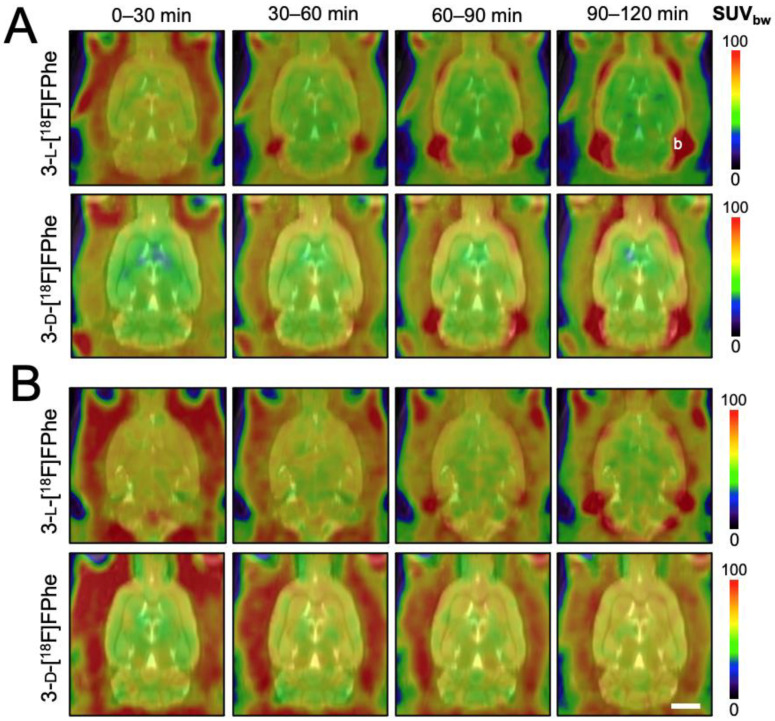
Biodistribution of 3-l-[^18^F]FPhe and 3-d-[^18^F]FPhe in the brain of healthy rats (*n* = 3 rats per tracer) determined without (**A**) and with (**B**) the peripheral aromatic l-amino acid decarboxylase inhibitor benserazide (15 mg/kg bw). Horizontal PET images displayed in time frames of 30 min each and projected onto an MRI template. The letter b in the upper right image indicates the lambdoidal crest, where the bone uptake was quantified. Scale bar: 5 mm.

In a first set of in vivo experiments, the brain accumulation and biodistribution of 3-[^18^F]FPhes in healthy rats was investigated using µPET. As illustrated in Figure 6, both tracers exhibited a relatively homogenous brain distribution.

As expected, the brain uptake of the l-enantiomer was significantly higher in animals pre-treated with the AADC inhibitor benserazide (F(27,112) = 27.09, *p* < 0.0001 for factor “timepoint”; 20–92 min p.i.: post hoc 0.001 < *p* < 0.05 and 102–120 min p.i.: post hoc 0.0200 < *p* < 0.05) when compared to the control group without benserazide pretreatment. In contrast, the brain uptake of 3-d-[^18^F]FPhe was higher without benserazide pretreatment, although the difference did not reach statistical significance. Based on time activity curves (TACs) and SUV_mean_ values, both tracers showed low accumulation in the lambdoidal crest, with no significant difference in bone uptake between 3-l-[^18^F]FPhe and 3-d-[^18^F]FPhe measured with or without the AADC inhibitor benserazide. However, in measurements with 3-l-[^18^F]FPhe, a progressive accumulation of radioactivity in bones was observed, which could reflect gradual defluorination of the tracer during the experiments. In experiments with 3-d-[^18^F]FPhe, the bone uptake of radioactivity remained static over time (Figure 6, Table 1).

**Figure 7:**
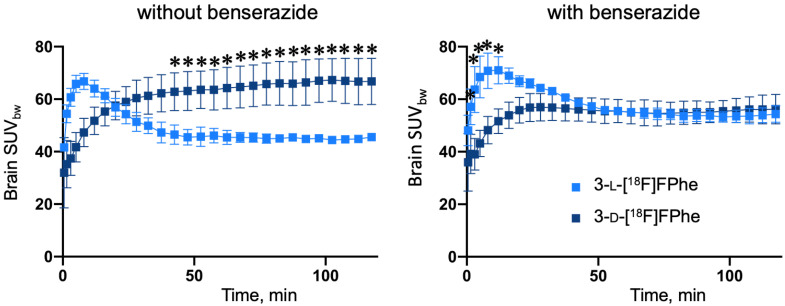
Comparison of brain time activity curves of 3-l- (light blue) and 3-d-[^18^F]FPhe (dark blue) in healthy rats (*n* = 3 each) with (**right**) and without (**left**) benserazide pretreatment. Significantly higher uptake of 3-d-[^18^F]FPhe at 42–120 min p.i. was observed in animals without benserazide pretreatment. * indicates significant differences between 3-l-[^18^F]FPhe and 3-d-[^18^F]FPhe.

Based on the TACs obtained in the absence of benserazide, the brain uptake of 3-l-[^18^F]FPhe showed an early peak followed by a relatively fast washout until it reached a static level at roughly 50 min p.i. (Figure 7), which is in accordance to the results of the in vitro cellular uptake experiments (Figure 3). In contrast, the brain uptake of 3-d-[^18^F]FPhe gradually increased over time and reached a significantly higher level at 42–120 min p.i. when compared to 3-l-[^18^F]FPhe (F(27,112) = 3.320, *p* < 0.0001 for factor “timepoint”; post hoc 0.0001 < *p* < 0.0217) (Figure 7). This effect was not observed after pretreatment with benserazide. In these experiments, the brain uptake of the l-isomer at early timepoints was significantly higher than that of the d-isomer, while comparable levels were reached at 50 min p.i. (Figure 7). Close inspection of the brain distribution also revealed a common uptake pattern for [^18^F]FET and 3-l-[^18^F]FPhe (Figure S1A–F), but not 3-d-[^18^F]FPhe. However, while [^18^F]FET exhibited a steadily increasing TAC (Figure S1G) with high uptake in the cortex, thalamus, and cerebellum being visible 90–120 min p.i., 3-l-[^18^F]FPhe showed the highest uptake during the first 30 min p.i., which was followed by a fast washout. As a consequence, the high uptake of 3-l-[^18^F]FPhe in cortex, thalamus, and cerebellum was already reached during this early period but lost in later timeframes.

In summary, both tracers showed a promising combination of high brain and tolerable bone uptake.

#### 3.3.2. Evaluation in Subcutaneous Tumor Xenograft Models

**Figure 8:**
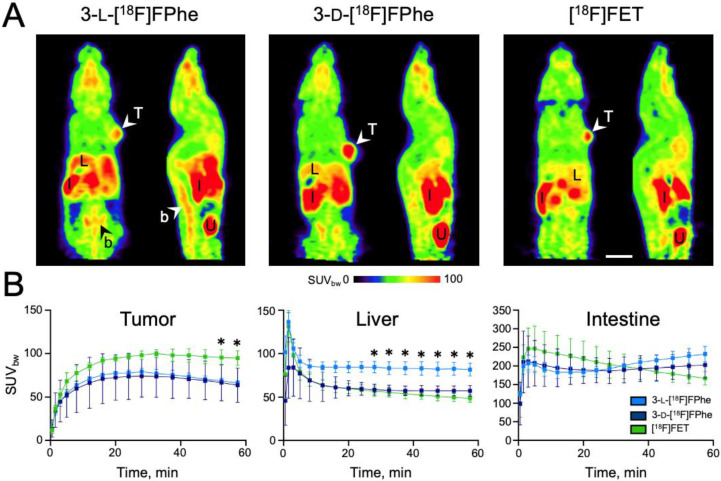
(**A**) Horizontal and sagittal non-Gauss filtered summed PET images (0–60 min p.i.) of MCF-7 tumor-bearing mice injected with the indicated PET tracers. Tumor cells were implanted subcutaneously in the right shoulder area. (**B**) Regional TACs (tumor, liver, intestine) of the different tracers (*n* = 3 mice per tracer). * indicates significant differences in uptake compared to [^18^F]FET. Abbreviations: b: bone, I: intestine, L: liver, T: tumor, U: urinary bladder. Scale bar: 1 cm.

Next, the tracer candidates were evaluated in tumor xenograft models based on the same cell lines used in the cellular uptake studies. To this end, PC-3 (human prostate adenocarcinoma) and MCF-7 (human breast adenocarcinoma) tumor cells were implanted subcutaneously in SCID mice. As illustrated in Figure 8, the subcutaneous MCF-7 tumor was clearly visualized by all three tracers. The lowest background accumulation was observed for 3-d-[^18^F]FPhe. The tumor-to-muscle ratios were highest for 3-d-[^18^F]FPhe both in MCF-7 (3-l-[^18^F]FPhe: 1.82 ± 0.08; 3-d-[^18^F]FPhe: 2.22 ± 0.07; [^18^]FET: 2.0 ± 0.11) and PC-3 tumors (3-l-[^18^F]FPhe: 1.79 ± 0.27; 3-d-[^18^F]FPhe: 2.7 ± 0.33; [^18^]FET: 2.12 ± 0.17). At the late timepoints, the MCF-7 tumor uptake of [^18^F]FET was significantly higher than that of 3-d-[^18^F]FPhe (F(15,96) = 18.11, *p* < 0.0001 for factor ‘timepoint’; 52–60 min p.i.: post hoc 0.0262 < *p* < 0.0439) and 3-l-[^18^F]FPhe (57 min p.i.: post hoc *p* < 0.0452). 3-l-[^18^F]FPhe demonstrated a significantly higher liver uptake at 28–60 min p.i. than [^18^]FET (F(15,112) = 15.22, *p* < 0.0001 for factor “timepoint”; post hoc 0.0092 < *p* < 0.0419). Tracer uptake kinetics obtained in animals bearing PC-3 tumor xenografts (*n* = 5 per tracer) were similar, but without significant differences for tumor uptake. Thus, the new tracers showed imaging properties on par with [^18^]FET when used for the visualization of subcutaneous tumors by PET.

#### 3.3.3. Evaluation in an Orthotopic Tumor Xenograft Model

**Figure 9:**
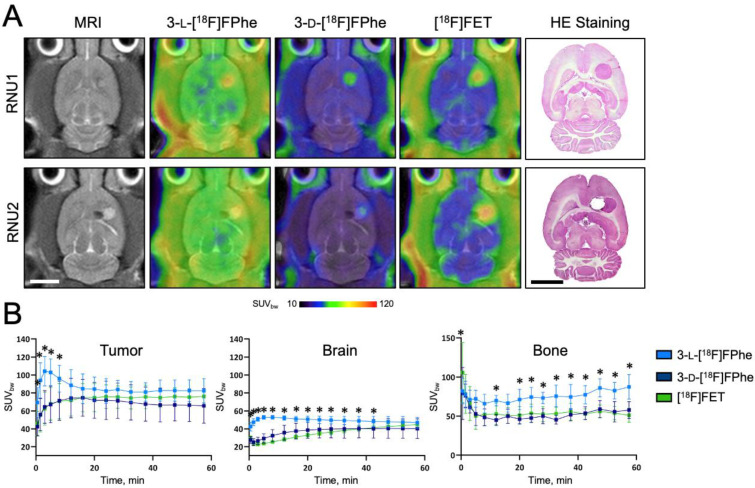
(**A**) Horizontal MRI and PET images (summed PET images from 0–60 min p.i. are projected onto the MRI images) of two rat brains (RNU1 and RNU2) with intracerebral U87 glioblastomas. Tumors were clearly delineated using all three tracers. The histological HE stainings performed post mortem are also shown. (**B**) Mean regional TACs of the different tracers (3-l-[^18^F]FPhe: *n* = 8, 3-d-[^18^F]FPhe: *n* = 6, [^18^F]FET: *n* = 9). 3-l-[^18^F]FPhe demonstrated the highest tumor uptake at early timepoints. * indicates significant differences in uptake compared to [^18^F]FET. Scale bars: 5 mm.

Next, an orthotopic xenograft rat model was used to evaluate the accuracy of the tracers for imaging intracerebral gliomas. As illustrated in Figure 9, intracerebral tumors induced by implantation of U87 glioblastoma cells were clearly delineated by all investigated tracers. At early timepoints, significantly higher tumor uptake of 3-l-[^18^F]FPhe was observed. Similarly to the biodistribution studies in healthy rats, the lower metabolic stability of 3-l-[^18^F]FPhe was reflected in a higher skull uptake of radioactivity (Figure 9B).

The tumor-to-brain (TBrR) ratio was calculated separately for 0–30 min and 30–60 min p.i.. We found a significant main effect of factor time with F(1,20) = 37.7; *p* < 0.0001, as well as a significant time × tracer interaction with F(2,20) = 9.2; *p* = 0.00015. Post-hoc testing revealed that [^18^F]FET showed a significantly higher TBrR when compared to 3-l-[^18^F]FPhe (*p* = 0.0065; Table 2).

**Figure 10:**
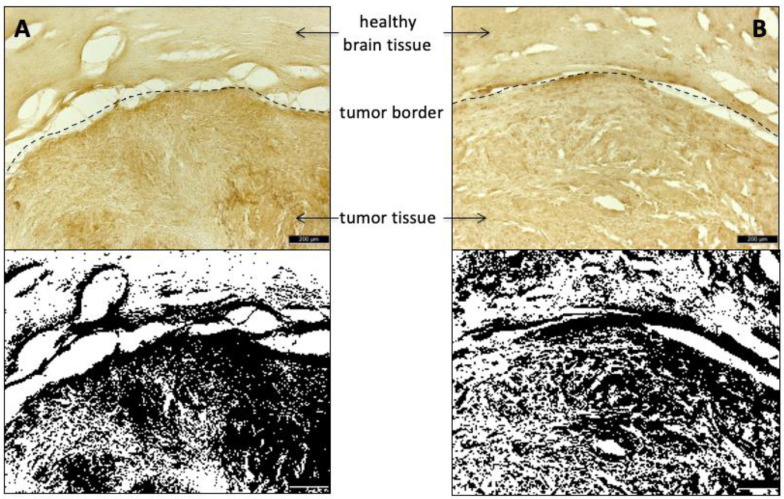
Immunohistochemical staining of tumor-containing brain sections with (**A**) anti-LAT1/SLC7A5 and (**B**) anti-ASCT2/SLC1A5 antibodies with the corresponding binary images (threshold: 50% of intensity). An increased expression of both amino acid transporters can be seen in the tumor tissue (LAT1: 59.0 ± 8.1% of area; ASCT2: 48.9 ± 5.8% of area) compared to the healthy brain tissue (LAT1: 22.4 ± 4.8% of area; ASCT2: 32.4 ± 5.6% of area). Scale bar: 200 µm.

The expression of LAT1 and ASCT2 transporters in the tumor tissue was confirmed by immunohistochemical analysis of the intracerebral tumors following the in vivo studies (Figure 10).

## 4. Discussion

In the present work, two enantiomeric 3-[^18^F]fluorophenylalanines were prepared and evaluated in vitro and in vivo to assess their potential as PET probes for tumor imaging, using [^18^F]FET as a benchmark tracer.

In vitro uptake of the new tracers in different tumor cell lines was found to be similar (U87 MG) or higher (MCF-7 and PC-3) than that of [^18^F]FET. Since Phe can serve as a substrate for various amino acid transport systems, we used different inhibitors to characterize the mechanisms for tumor uptake of 3-[^18^F]FPhes in more detail. As expected, the most pronounced effect was observed after competitive inhibition of amino acid transport system L with BCH, which produced a significant, concentration-dependent decrease in cellular uptake. However, as BCH also affects the Na^+^-dependent transport systems B^0^ and B^0,+^, a contribution of these systems to tracer uptake cannot be excluded without further studies. It is usually assumed that [^18^F]FET can be transported via system L, B^0^, and B^0,+^, with the majority of transport being mediated by LAT1 and, to a lesser extent, LAT2, two structurally similar transporters belonging to system L [3,6,28]. LAT3 and LAT4, two other known members of system L, have also been shown to transport Phe [9], although the role of LAT4 for carcinogenesis is still ambiguous [29,30]. In any case, since suitable LAT3 and LAT4 inhibitors are still lacking, the role of these transporters was not assessed in the present work and remains to be elucidated in future studies. It has been proposed that the ASC system may also play a role for transport of [^18^F]FET into brain tumor tissues [31], which is in line with our finding that competitive inhibition of this system with l-serine significantly decreased the uptake of [^18^F]FET as well as [^18^F]FPhes. ASC transporters, as well as system L transporters, belong to the family of solute carriers (SLC) with two transporters (ASCT1/SLC1A4 and ASCT2/SLC1A5) specific for neutral amino acid substrates [32].

Remarkably, the inhibition of either system L or system ASC almost completely prevented cellular uptake of all three radiolabeled amino acids. Based on previous studies demonstrating sufficient selectivity of the applied inhibitors [33,34,35], this suggests that tracer uptake into tumors may critically depend on an interplay of both transporter systems. According to the so-called “tertiary active transport model,” LAT1 uses the intracellular pool of small neutral amino acids generated by ASCT2 transport activity for exchange with large, essential amino acids from the extracellular space [36]. In this view, inhibition of the ASC system could indirectly reduce uptake mediated by LAT1, which is in line with the results of our cell uptake studies that systems L and ASC, but not amino acid transporter system A, are required for uptake of all three tracers into the tumor cells. The observation that both system L and system ASC play an important role in transporting 3-l-[^18^F]FPhe and 3-d-[^18^F]FPhe could help to develop Phe-based antitumoral agents or prodrugs in the sense of a dual targeting treatment. Given that a double knockout of LAT1 and ASCT2 might be lethal for cancer cells [32], a specific inhibition of these transporters confined to the tumor may represent a promising therapeutic approach.

The brain radioactivity uptake observed in the in vivo experiments with healthy rats demonstrates that both novel tracers are efficiently transported across the blood-brain-barrier (BBB). This is in line with the assumption that LAT1, which is typically regarded as responsible for the brain uptake of amino acids such as Phe and [^18^F]FET, shows no stereoselectivity with regard to its substrates and can also transport d amino acids, as already noted in a number of recent publications [20,37]. As discussed in more detail below, the fast component of brain uptake in healthy rats, which was observed for 3-l- but not 3-d-[^18^F]FPhe, could be related to LAT2, which is also involved in amino acid transfer across the BBB but was reported to only transport l amino acids [38]. As expected based on the stereospecificity of AADC, the peripheral inhibition of this enzyme by benserazide significantly increased the brain uptake of 3-l- but not 3-d-[^18^F]FPhe in healthy rats. Thus, in the absence of benserazide, peripheral AADC would be expected to selectively metabolize 3-l-[^18^F]FPhe into [^18^F]fluorophenylethylamine, which may be subject to rapid degradation by monoaminoxidases and other enzymes.

When PET scans were performed in subcutaneous mouse and orthotopic rat tumor xenograft models, the new tracers showed imaging properties on par with [^18^F]FET, except for a higher tumor uptake of [^18^F]FET in the subcutaneous MCF-7 model at late timepoints and a higher TBrR of [^18^F]FET compared to 3-l-[^18^F]FPhe in the orthotopic U87 glioma model at early timepoints. As expected based on its stereochemistry, which should prevent protein incorporation, 3-d-[^18^F]FPhe showed a lower background activity, even though there was no statistically significant difference when compared to the other two tracers. Interestingly, the distinct tumor cell uptake behavior of 3-l-[^18^F]FPhe compared to the other tracers observed in vitro was only partly reflected in the in vivo experiments. Thus, similar to the in vitro experiments, the in vivo uptake of 3-l-[^18^F]FPhe into intracerebral U87 glioblastomas was characterized by a rapid but transient rise to significantly higher levels, followed by a decline to levels comparable with the other two tracers at roughly 20 min p.i.. As a consequence, the characteristic uptake pattern seen with [^18^F]FET at 90–120 min p.i. was achieved much earlier (0–30 min) with 3-l-[^18^F]FPhe. In contrast, the time course of 3-l-[^18^F]FPhe uptake into subcutaneous MCF-7 tumors was essentially the same as that of other two tracers. This observation could indicate that not cellular uptake per se but some slower process (such as transport to the tumor) is the rate-limiting step for tumor accumulation of 3-l-[^18^F]FPhe in this model. In any case, the higher initial tumor uptake of 3-l-[^18^F]FPhe in the glioblastoma model deserves further investigation, as it could possibly be exploited to shorten the scan time of brain tumor patients in the future. Interestingly, several previous studies in patients have found the kinetics of [^18^F]FET uptake in high-grade gliomas to be characterized by an early peak and subsequent decline as well, while TACs in low-grade gliomas have shown a progressive increase more similar to the time course of [^18^F]FET uptake we observed in the orthotopic tumor model [39,40,41]. It will therefore be of interest to examine if the uptake kinetics of [^18^F]FPhes are also affected by the exact tumor grade and could be used to distinguish low-grade from high-grade gliomas.

With regard to the mechanism underlying the faster initial uptake of 3-l-[^18^F]FPhe, our data are insufficient for firm conclusions, but several observations argue for a more efficient transport of 3-l-[^18^F]FPhe by the relevant amino acid transporters, most notably LAT1 and/or LAT2. First, as already mentioned, the initial in vitro uptake of 3-l-[^18^F]FPhe into all three tumor cell lines examined was much faster and significantly higher than uptake of the other two tracers. Second, even though some degree of protein-incorporation of 3-l-[^18^F]FPhe could be demonstrated in vitro, it seems unlikely that this was responsible for the high initial tumor uptake, since no significant differences in tumor uptake of 3-l-[^18^F]FPhe compared to [^18^F]FET and 3-d-[^18^F]FPhe were observed at later timepoints. Third, the brain uptake of 3-l-[^18^F]FPhe in healthy rats, which is likely to be primarily mediated by LAT1 and LAT2 [42,43], showed almost the same time course as the tumor uptake of this tracer in the glioblastoma model. In this regard, a potential explanation for the faster brain and tumor uptake kinetics of 3-l- compared to 3-d-[^18^F]FPhe could be the contribution of LAT2, which has been reported to transport only l amino acids [38]. As such, overexpression of the amino acid transporters rather than increased incorporation into proteins seems to be primarily responsible for a high tracer accumulation in tumor tissue. Increased expression of LAT1 and ASCT2 in tumor tissue compared to healthy brain tissue was confirmed by immunohistochemistry, which further supports this hypothesis. However, the extent to which other members of the L and ACS systems are involved remains to be elucidated. The mechanism responsible for the fast partial washout of 3-l-[^18^F]FPhe remains unclear as well, but it could be related to the fact that amino acid transport by system L can occur in both directions. As such, accumulated 3-l-[^18^F]FPhe that is neither bound nor metabolized could, in part, be lost by exchange transport via LAT2 or another bidirectional amino acid transporter. Passive efflux of 3-l-[^18^F]FPhe via the disrupted BBB could possibly provide an alternative explanation for the washout. This, however, seems unlikely, given that significant efflux: (i) of 3-l-[^18^F]FPhe from tumor cells was also observed in the in vitro experiments, (ii) of 3-l-[^18^F]FPhe from brain was also observed in healthy rats, and (iii) of [^18^F]FET and 3-d-[^18^F]FPhe was neither observed in the in vitro experiments nor in the orthotopic tumor model.

## 5. Conclusions

In summary, the two novel fluorinated phenylalanine tracers evaluated in the present study are well suited for the visualization of even small solid tumors. Based on direct comparison with the established tracer [^18^F]FET, certain advantages, such as reduced background activity (3-d-[^18^F]FPhe) and significantly higher early tumor uptake in an orthotopic glioblastoma model (3-l-[^18^F]FPhe), justify further evaluation of both tracers.

## Figures and Tables

**Table 1:** Brain and bone uptake of 3-l-[^18^F]FPhe and 3-d-[^18^F]FPhe in healthy rats quantified by SUV_bw_ (mean ± SD) 30–60 min post injection. No significant differences were seen for the comparison “with benserazide” versus “without benserazide”: F(1,8) = 27.98, *p* < 0.007, Sidak’s post hoc *p* = 0.26 and *p* = 0.69.

Model	Region	3-l-[^18^F]FPhe	3-d-[^18^F]FPhe
with benserazide	Brain SUV_mean_ Bone SUV_mean_	59.31 ± 0.68 68.73 ± 3.95	56.71 ± 3.19 86.27 ± 11.71
without benserazide	Brain SUV_mean_ Bone SUV_mean_	53.41 ± 4.73 79.80 ± 6.61	63.19 ± 4.98 84.65 ± 8.36

**Table 2:** Tumor-to-brain-ratios (TBrR) and tracer uptake in the orthotopic rat glioma model quantified by SUV.

Parameter	Region	3-l-[^18^F]FPhe	3-d-[^18^F]FPhe	[^18^F]FET
SUV_max_	tumor	107.62 ± 11.29	86.00 ± 4.31	90.15 ± 7.66
SUV_mean_	brain	47.20 ± 2.56	36.67 ± 1.47	34.02 ± 1.55
TBrR 0–60 min	tumor/brain	2.28 ± 0.19	2.33 ± 0.20	2.65 ± 0.04
TBrR 0–30 min	tumor/brain	2.31 ± 0.38	2.55 ± 0.49	2.93 ± 0.62 *
TBrR 30–60 min	tumor/brain	2.25 ± 0.44	2.14 ± 0.32	2.29 ± 0.28

* Significant difference between [^18^F]FET and 3-l-[^18^F]FPhe (see text).

## Data Availability

The data presented in this study are available on reasonable request from the corresponding author.

## Supplementary Materials

The following are available online at https://www.mdpi.com/article/10.3390/cancers13236030/s1, Figure S1: Brain uptake pattern of 3-l-[^18^F]FPhe and [^18^F]FET.
