# Supplementary figures and images for: Evaluation of 3-l- and 3-d-[18F]Fluorophenylalanines as PET Tracers for Tumor Imaging
Source: Cancers (Basel). 2021 Nov 30;13(23):6030. doi: 10.3390/cancers13236030 (PMC8656747; doi:10.3390/cancers13236030)

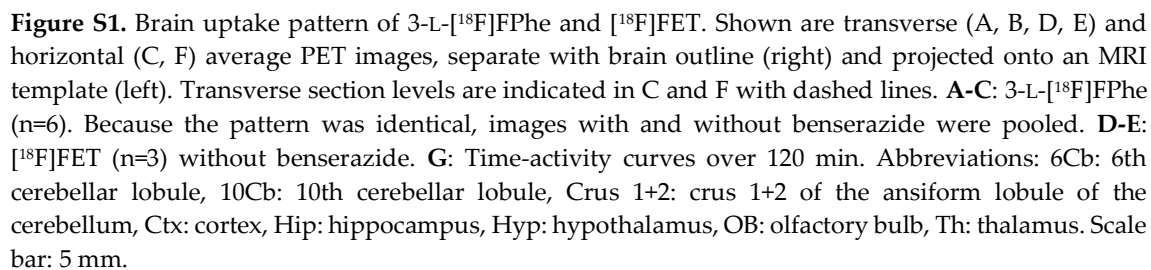

Supplement: Supplementary file 1 [file cancers-13-06030-s001.zip › cancers-1455995-supplementary.pdf]
